# *Drosophila melanogaster* as a model to study polymicrobial synergy and dysbiosis

**DOI:** 10.3389/fcimb.2023.1279380

**Published:** 2023-12-21

**Authors:** Xixi Cao, Jessica Scoffield, Baotong Xie, David B. Morton, Hui Wu

**Affiliations:** ^1^Department of Integrative Biomedical & Diagnostic Sciences, Oregon Health and Science University School of Dentistry, Portland, OR, United States; ^2^Department of Microbiology, School of Medicine, University of Alabama at Birmingham, Birmingham, AL, United States

**Keywords:** bacterial virulence, biofilms, polymicrobial interactions, *Drosophila*, Streptococcus

## Abstract

The fruit fly *Drosophila melanogaster* has emerged as a valuable model for investigating human biology, including the role of the microbiome in health and disease. Historically, studies involving the infection of *D. melanogaster* with single microbial species have yielded critical insights into bacterial colonization and host innate immunity. However, recent evidence has underscored that multiple microbial species can interact in complex ways through physical connections, metabolic cross-feeding, or signaling exchanges, with significant implications for healthy homeostasis and the initiation, progression, and outcomes of disease. As a result, researchers have shifted their focus toward developing more robust and representative *in vivo* models of co-infection to probe the intricacies of polymicrobial synergy and dysbiosis. This review provides a comprehensive overview of the pioneering work and recent advances in the field, highlighting the utility of *Drosophila* as an alternative model for studying the multifaceted microbial interactions that occur within the oral cavity and other body sites. We will discuss the factors and mechanisms that drive microbial community dynamics, as well as their impacts on host physiology and immune responses. Furthermore, this review will delve into the emerging evidence that connects oral microbes to systemic conditions in both health and disease. As our understanding of the microbiome continues to evolve, *Drosophila* offers a powerful and tractable model for unraveling the complex interplay between host and microbes including oral microbes, which has far-reaching implications for human health and the development of targeted therapeutic interventions.

## Introduction

*Drosophila melanogaster* or the “fruit fly”, a simple invertebrate, is becoming an attractive model organism for studying a wide range of topics of complex human biology because it shares significant biological similarity to human systems, and has tractable genetic manipulating tools (Chao et al., 2017). In addition, fly stocks and databases are publicly available and accessible (https://flybase.org/). Importantly, 75% of human genes related to disease have their homologs identified in fruit flies (Yamaguchi and Yoshida, 2018; Cagan et al., 2019), which enables the robust and in-depth study of gene regulation, protein interactions and posttranslational modification of conserved human homologs, and their impact on pathogenesis. Successful transformation of our basic understanding of fruit fly biology to the discovery of new genetic disorders in human disease has promoted the recent expansion of employing *Drosophila* to model human diseases, which will accelerate the scientific discovery of new signaling and metabolic pathways related to human disease and facilitate the translation of the basic science to clinical applications. The flies are permissive to infection by diverse species of microorganisms that often cause diseases in humans, and as such, is an attractive model for studying bacterial virulence and pathogenesis (Edwards and Kjellerup, 2012; Buchon et al., 2014). Several features of *D. melanogaster* make it an attractive model to study bacterial interactions, including simple endogenous microbial community, high-throughput screening potential, genetic tractability, cost-effectiveness, and ability to recapitulate key virulence events such as *in vitro* biofilm formation in microbial colonization of the fly’s crop. The fly crop is found to be the most common anatomic site where bacteria (both stable endogenous species coevolved with flies and temporarily or transiently infected bacteria) reside, which represents an excellent accessible port to explore microbial-microbial interactions as well as the microbial-host interactions. Furthermore, fruit flies are readily adapted to their diets and develop a correlative microbial community, which offers a unique opportunity to determine how nutrients (such as dietary sugars) and other environmental conditions interact with the host genetic components to shape a new polymicrobial landscape that may contribute to host physiology and pathology. In some instances, the development of fly microbiota directly modulates host health and disease (Gould et al., 2018; Brinker et al., 2019), which offers great opportunities to dissect underlying mechanisms.

## Polymicrobial interactions of oral microbes in *Drosophila melanogaster*

**Figure 1:**
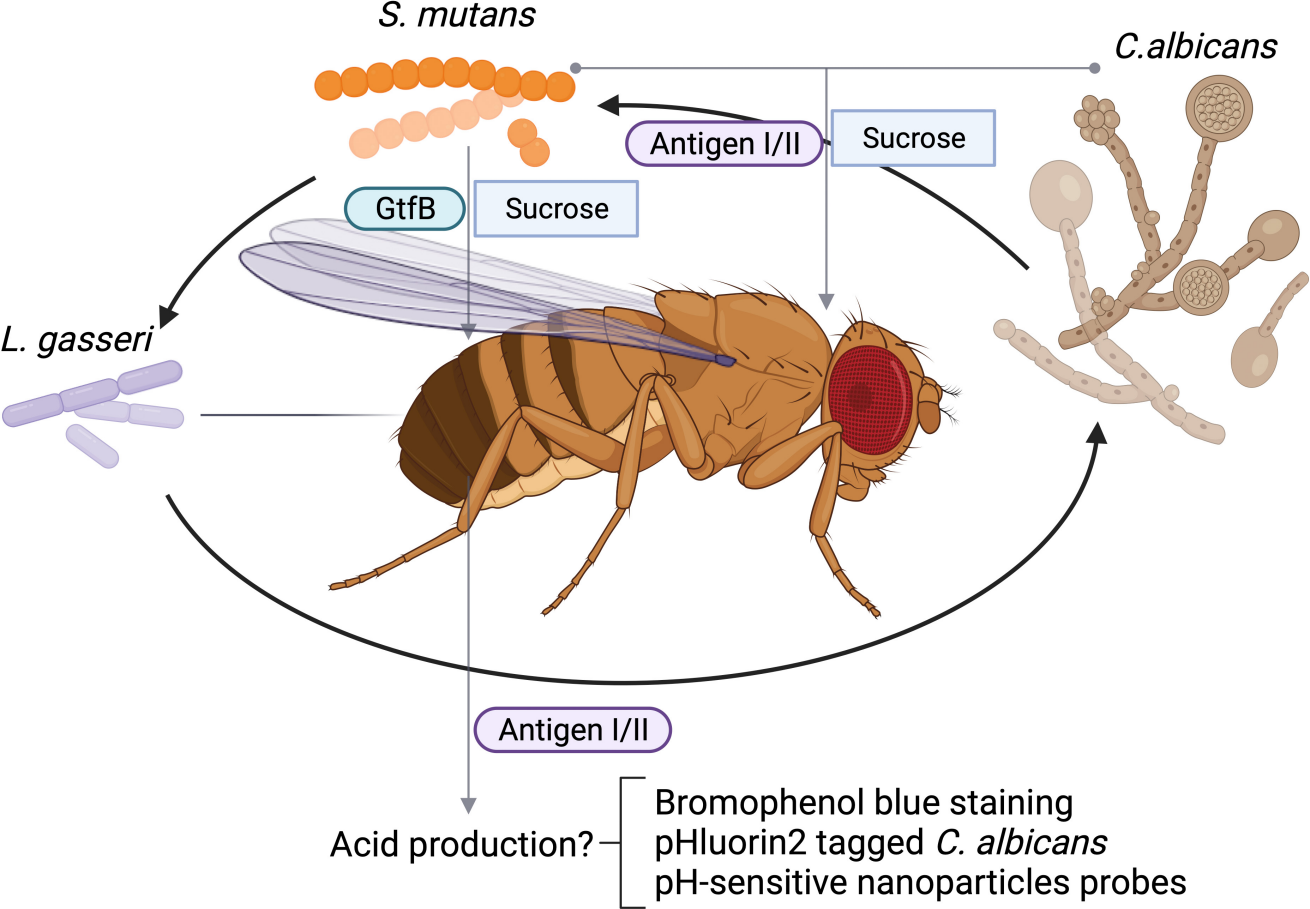
Schematic polymicrobial interactions of oral microbes in *D. melanogaster*. Acidic condition from *L. gasseri* fed or *Streptococcus mutans* and *Candida albicans* coinfected can be tracked by bromophenol blue staining, an acidic staining, or monitored by fluorescent microscopic examination of labeled *C. albicans*. *C. albicans* can be labeled with phlourin2 pH sensor (*C. albicans*-PHL2). Antigen I/II mediated interactions between *C. albicans* and and acidic condition can be monitored.

Biofilm formation is important for the fitness and virulence of many diverse microbial species especially oral microbes. The *Drosophila* crop serves as a reservoir for bacterial colonization, and therefore, is used to assess both single and multi-species biofilms. The oral feeding model of *D. melanogaster* has been used to study microbial interactions that are critical for biofilm formation among various oral microorganisms. One of advantage of using the feeding model to study oral microbial interactions is that the model utilizes sucrose, which is an important substrate that facilitates the attachment of microbes to the tooth surface. It has been well documented that sugary diets disrupt symbiotic microbial community in the oral cavity and promote dysbiosis such as a cariogenic community. *Streptococcus mutans* forms robust, sucrose-dependent biofilms on the tooth surface, colonizes the oral cavity, and becomes a major contributor of dental caries. Infection of *Drosophila* with *S. mutans* using the sucrose feeding protocol reveals that the colonization of *S. mutans* in the fly depends on a key biofilm matrix enzyme GtfB (Peng et al., 2016b), an important virulence component in humans and in rodent caries model, demonstrating the validity of this model. Like many microbial infections, dental caries is a polymicrobial infection. In addition to contribution by various bacteria, an opportunistic fungal organism *Candida albicans* is commonly co-isolated with *S. mutans* from plaque in children with severe early childhood caries, and these two species display synergism in worsening disease progression of dental caries in a rodent model (Bowen et al., 2018). Using the oral feeding model of *Drosophila*, the polymicrobial synergy between *C. albicans* and *S. mutans* is recapitulated. In addition, this model enables the rapid characterization of the specific microbial interaction and identification of microbial virulence factors involved. The *S. mutans* surface protein antigen I/II, a well-studied adhesin that is not required for single species biofilm formation of *S. mutans* under sucrose condition, is found to be required for the enhanced colonization of *C. albicans* in the polymicrobial setting during co-infection of *Drosophila melanogaster *(Yang et al., 2018) (**Table 1**). The *in vivo* results in *Drosophila* mirror results generated in both *in vitro* studies and *in vivo* experiments using a rat model of dental caries, suggesting the *Drosophila* model can be a reliable tool to examine microbial interactions and uncover new insights into microbial colonization in the oral cavity. Furthermore, the *in vitro* co-culture under the cariogenic condition (1% sucrose) increases acid production, a hallmark of cariogenic property. The fly model can be readily used to explore this phenotype as the flies gut is known to be acidic, and tolerate acids, and the pH-sensitive dye bromophenol blue can be used to monitor pH changes in the flies’ gut (Iatsenko et al., 2018). We propose this labeling method is feasible in tracking pH changes occurred in the flies’gut, We envision that infection of flies with lactate producing bacteria, such as *Lactobacillus gasseri* would enable its labeling by bromophenol blue staining. *L. casei* promotes biofilm formation when co-cultured with *S. mutans* (Wen et al., 2017). The application of this probe will allow us to determine the synergistic interaction between *L. gasseri* and *S. mutans*, or *C. albicans* and *S. mutans in vivo* in flies, and evaluate whether antigen I/II is required for the increased acid production during the process of the co-colonization of *S. mutans* and *C. albicans* in the flies’ gut as illustrated (**Figure 1**). Alternatively engineering recombinant microbes with pH-sensitive fluorescent probes could provide an invaluable tool to monitor changes in extracellular pH in real time during co-infection of flies (Tournu et al., 2017). It is well-documented that co-culture of *S. mutans* and *C. albicans* synergistically increases acid production *in vitro*, which can be tracked by the use of a recombinant *C. albicans* strain tagged with pHluorin2, a pH-sensitive variant of green fluorescent protein engineered by Dr. Palmer’s group (Tournu et al., 2017) as illustrated (**Figure 1**). The use of the pH-sensitive strain should facilitate noninvasive analysis of multispecies biofilm-associated pH change *in vivo* and help develop a robust *in vivo* screen to determine how other oral bacteria interact with *C. albicans* and manipulate cellular and environmental pH values, a key cariogenic virulence factor (Yang et al., 2018). Future studies will be focused on the development of more robust and versatile probes that are able to report dynamic pH changes during co-infection. In this regard, there are numerous pH-sensitive nanoparticles reported that can be explored in the study of contribution of polymicrobial interactions to pH dynamics (Fulaz et al., 2019; Kromer et al., 2022).

**Table 1:** Microbes studied in Polymicrobial interactions in *D. melanogaster*.

Species	Locations	Diseases	Mechanisms of Colonization	References
*Streptococcus mutans*	Dental plaque	Dental caries	Biofilm matrix-dependent colonization of *S. mutans*	Peng et al., 2016b
*Candida albicans*	Dental plaque in children with severe early childhood caries	Dental caries	*A*ntigen I/II enhanced co-colonization of *S. mutans* & *C. albicans*	Yang et al., 2018
*Streptococcus parasanguinis*	Oral cavity	associated with a healthy microbiota	*A. actinomycetemcomitans* mediated enhanced colonization of *S. parasanguinis*	Duan et al., 2016
*Aggregatibacter actinomycetemcomitans*	gingival and periodontal pocket sites in adolescents	Aggressive periodontitis	*S. parasanguinis* mediated inhibition of *A. actinomycetemcomitans* colonization	Duan et al., 2016
*Streptococcus salivarius*	Oral cavity and upper respiratory tract	Opportunistic pathogen	carbohydrate binding-mediated colonization of *S. salivarius* and *P. aeruginosa*	Stoner et al., 2022
*Staphylococcus aureus*	upper respiratory tract and on the skin	Human opportunistic pathogen	eDNA binding-mediated enhanced *S. aureus* colonization by *S. parasanguinis* Enhanced virulence by co-infections of *Staphylococcus spp* with *P. aeruginosa*	Wang et al., 2023, Sibley et al., 2008
*Pseudomonas aeruginosa*	Lung	Cystic fibrosis	Adhesins-mediated enhanced colonization of *S. parasanguinis* by *P. aeruginosa via its* alginate production *Attenuation of P. aeruginosa* virulence by *S. parasanguinis* in the presence of nitrite The nitrite reductase mediated *P. aeruginosa* survival during co-infection with *S. parasanguinis*;	Scoffield et al., 2017, Scoffield and Wu, 2015, Scoffield and Wu, 2016

The *Drosophila* model has also been used to discern interactions between hydrogen peroxide-producing oral commensal *Streptococcus parasanguinis* and periodontal pathogen *Aggregatibacter actinomycetemcomitans*. *A. actinomycetemcomitans* establishes localized aggressive periodontitis in gingival and periodontal pocket sites in adolescents (Fine et al., 2007). Interestingly, the occurrence of *S. parasanguinis* at subgingival sites with *A. actinomycetemcomitans* and another oral bacterium is associated with increased bone loss in humans (Fine et al., 2013). Studies evaluating the synergistic relationship between the aforementioned commensal and periopathogen have discovered that *A. actinomycetemcomitans* promotes the biofilm formation of *S. parasanguinis* and tightly controls production of hydrogen peroxide by *S. parasanguinis.* Low concentrations of hydrogen peroxide dose dependently modulate the biofilm formation of *S. parasanguinis.* These interesting *in vitro* findings are readily recapitulated *in vivo* during co-infection of *Drosophila* with *A. actinomycetemcomitans* and *S. parasanguinis*, colonization of *S. parasanguinis* is significantly increased while the *A. actinomycetemcomitans* numbers are dramatically inhibited (Duan et al., 2016). Although the underlying mechanisms of inhibition of *A. actinomycetemcomitans*, and promotion of *S. parasanguinis* are not entirely clear currently, these results suggest that microbial interactions are more dynamic and complex than previously thought. The fly model provides a powerful tool to investigate complex polymicrobial infections observed in clinic, which should facilitate future in-depth mechanistic studies.

Additionally, oral commensal *S. parasanguinis* often shares the same ecological niche as *Staphylococcus aureus*. Using the fly model of colonization, we have determined that *S. parasanguinis* significantly increased *S. aureus* biofilm formation and enhanced its colonization *in vivo* in flies, which highlights how oral commensals may affect the fitness and persistence of *S. aureus* (Wang et al., 2023). It was determined that streptococcal biofilm-associated protein, BapA1, was essential for dual-species biofilm formation through its colocalization with staphylococcal extracellular DNA, emphasizing the significance of the interspecies biofilm matrix formed between streptococcal BapA1 and staphylococcal eDNA in the formation of polymicrobial interactions. The fly model not only allows mechanistic studies but also provides valuable insights that could inform future research and the development of novel therapeutic approaches targeting polymicrobial biofilms.

## Oral streptococci and *Pseudomonas aeruginosa* interactions in *Drosophila melanogaster*

*Drosophila* serves as a natural-route for polymicrobial infection or colonization, and could reflect the synergetic interactions between different species found in *in vitro* studies or clinical settings (Peters et al., 2012; Gould et al., 2018). Particularly, the current fly feeding model is regarded as a viable platform as it resembles a chronic infection. The localized infection and slow killing kinetics observed following oral infection of *Drosophila* by *P. aeruginosa* are consistent with the findings that *P. aeruginosa* forms micro-colonies or biofilms in the *Drosophila* crop (Mulcahy et al., 2011). Based on this established single infection model, *Drosophila* was explored as a tool to screen the interaction of *P. aeruginosa* with 40 diverse oropharyngeal streptococcal isolates that display varying synergism with *P. aeruginosa.* High-throughput screens using *Drosophila* survival as an outcome readout is simple and effective, which enables the identification of specific streptococcal species that potentiate or attenuate *P. aeruginosa* pathogenesis. Many streptococcal isolates are found to significantly promote the killing of flies by *P. aeruginosa*, suggesting the interactions between commensal oral streptococci and *P. aeruginosa* enhance virulence. Such findings are also observed *in vivo* as the presence of similar organisms in cystic fibrosis (CF) airways associates with the worsened outcome of an existing infection or a newly established infection with *P. aeruginosa* (Sibley et al., 2008). Intriguingly, opposite effects on virulence by polymicrobial interactions are also documented. Biofilm formation by an oral commensal *S. parasanguinis* is promoted by *P. aeruginosa*. *P. aeruginosa* causes chronic lung infections in CF patients and is a leading contributor of morbidity and mortality in CF disease. Consistently, an oral commensal, *S. parasanguinis* is commonly found in the CF lung and is associated with improved lung function (Filkins et al., 2012). We have used the *Drosophila* model to examine whether *S. parasanguinis* potentially drives positive health outcomes seen in CF lung infections by its interference with the pathogenesis of *P. aeruginosa*. *S. parasanguinis* surface adhesins, BapA1 and Fap1, mediate the enhanced colonization of *S. parasanguinis*, which is dependent on alginate production by *P. aeruginosa* (Scoffield et al., 2017). The binding of *S. parasanguinis* to alginate using surface adhesins may explain a potential mechanism of how this bacterium gets incorporated into the CF lung. In fact, another oral streptococcus frequently found colocalized with *P. aeruginosa*, *Streptococcus salivarius* employs a carbohydrate binding protein to interface with a different exopolysaccharide of *P. aeruginosa* to establish its colonization by promoting biofilm formation *in vitro* and in a *D. melanogaster* co-infection model(Stoner et al., 2022). Moreover, *S*. *parasanguinis* in the presence of nitrite protects *Drosophila* from killing by *P. aeruginosa* during co-infections (Scoffield and Wu, 2015), and the nitrite reductase activity by *P. aeruginosa* is required for the survival of this pathogen during co-infection with *S. parasanguinis* (Scoffield and Wu, 2016), suggesting a new nitrite-dependent anti-infective mechanism by the oral commensal *S. parasanguinis*. Interestingly, this mechanism is also relevant to the polymicrobial interaction between commensal *S. parasanguinis* and a cariogenic bacterium *S. mutans*. The presence of nitrite enables the hydrogen peroxide producing *S. parasanguinis* to inhibit *S. mutans* growth and biofilm formation *in vitro*. *S. parasanguinis* effectively competes with *S. mutans* within the nitrite containing two-species biofilm and inhibits production of a key biofilm matrix, glucans by *S. mutans in vitro.* These *in vitro* findings are further validated in an *in vivo* rat caries model. Commensal *S. parasanguinis* significantly inhibits cariogenic virulence induced by *S. mutans* when animals fed nitrite in the drinking water, in comparison with co-infected animals that received no nitrite (Scoffield et al., 2019). These laboratory studies support the clinical observation that high amounts of salivary nitrite are associated with lower prevalence of dental caries (Hohensinn et al., 2016). The protective mechanism offered by commensal streptococci and the host metabolite nitrite provides a potential novel therapeutic strategy by simply modulating dietary nitrite concentrations in the oral cavity.

## *Pseudomonas aeruginosa* and *Staphylococcus aureus* synergism in *Drosophila melanogaster*

*P. aeruginosa* and *S. aureus* co-infections are common in CF airway disease, suggesting there is a synergistic interaction between two organisms. Indeed, coinfection of animals with *P. aeruginosa* and *S. aureus* enhances virulence in two models, a mouse chronic wound model and a rat lung infection model. Such interactions are reproducible in a *Drosophila* infection model (Korgaonkar et al., 2013). In the oral feeding model, *P. aeruginosa* kills *Drosophila* within a few days, and *Staphylococcus spp*. itself does not kill flies. However, co-infections of *Staphylococcus spp* with *P. aeruginosa* increases the killing of flies dramatically while currently decreases the numbers of *Staphylococcus spp* by 10,000 fold, revealing a synergistic interaction in promoting *P. aerugonisa* virulence. More interestingly, the coinfection significantly upregulates the expression of genes responsible for *Drosophila* innate immunity, including various antimicrobial peptides (Sibley et al., 2008). Such synergistic enhancement of innate immunity is not evident using another fly infection model, the septic injury infection, suggesting the oral feeding model is uniquely suitable for the study of co-infection mediated synergy in innate immunity. Mechanistically, *P. aeruginosa* responds to the major surface component of *Staphylococcus aureus*, peptidoglycan via a two-component response regulator PA0601, which in turn triggers expression of a host of virulence factors including pyocyanin and elastase. Those virulence factors modulated by the quorum sense can kill both prokaryotic and eukaryotic cells. Importantly, the co-infection enhanced virulence demonstrated in *Drosophila* oral feeding model recapitulates the outcomes from a murine infection model. The engagement of microbial response regulator in the synergistic interaction is also illustrated in the fly model. In addition, the fly model allows the determination of the requirement of the peptideoglycan signaling in the inhibition of *S. aureus* growth by *P. aeruginosa*, which is also readily validated in murine chronic wound model (Korgaonkar et al., 2013). These data further support the notion that *Drosophila* can be employed as a surrogate host for polymicrobial infections, which offers an effective and efficient alternative to develop new therapeutics targeting polymicrobial interactions, synergistic activation of innate immunity, and numerous polymicrobial cues evident in the model in addition to providing new insights into understanding virulence and pathogenesis of *P. aeruginosa* in the polymicrobial infections.

## Microbial interactions in *Drosophila* modulate host physiology and pathology

**Figure 2:**
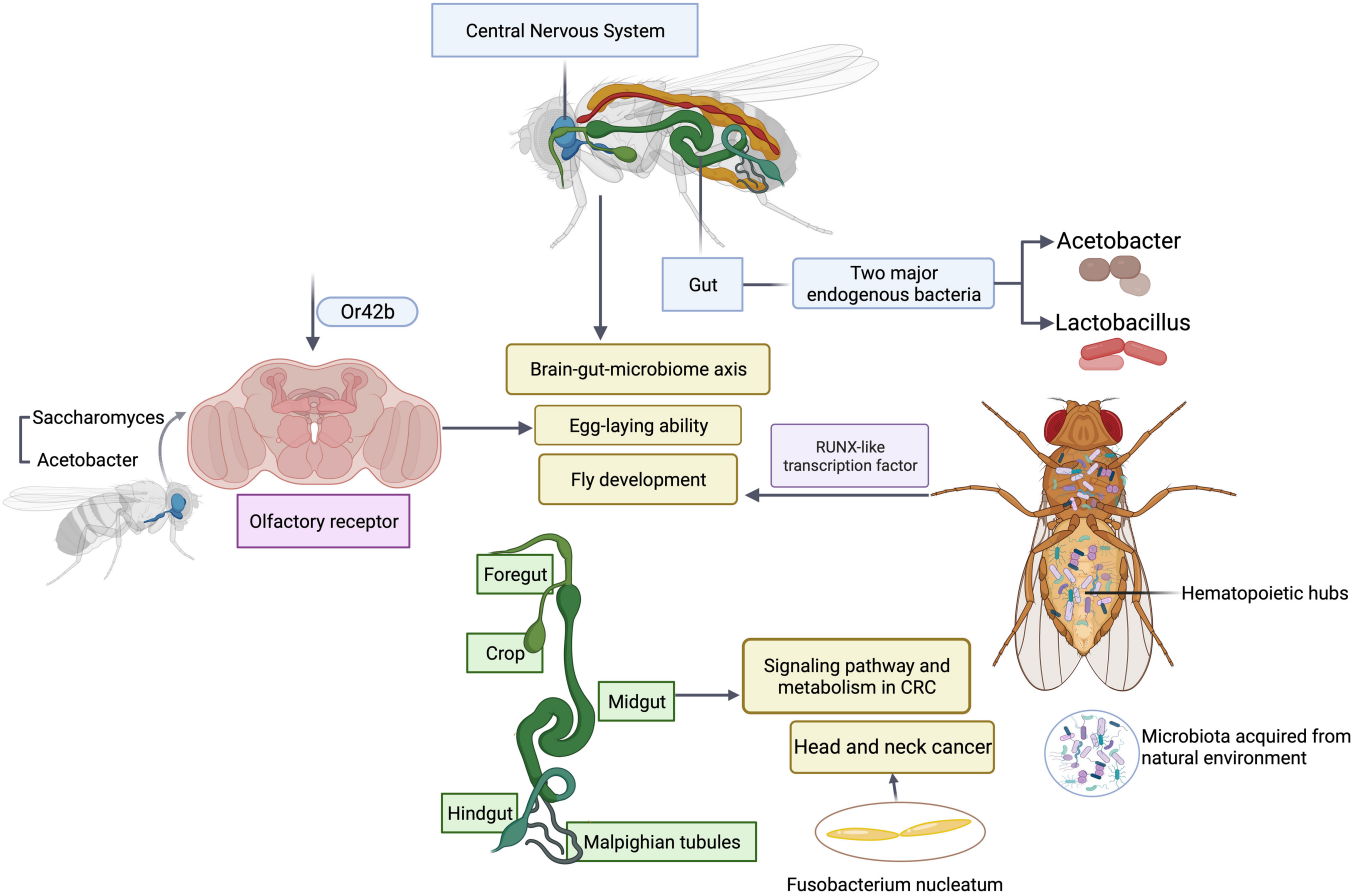
Microbial interactions in *Drosophila* modulate host physiology and pathology. Polymicrobial infections have a wide range of impacts on gut, central nervous system, olfactory organ and hematopoietic hubs of flies.

One advantage of using *Drosophila* as a model is its relative simple gut microbiota (Trinder et al., 2017). The low microbial diversity allows the investigators to systemically determine whether and how microbial function contributes to the development and maturation of host traits (Gould et al., 2018; Brinker et al., 2019). Bacteria metabolize various nutrient ingredients used by flies. Colonization of axenic (microbiota-free) flies with different groups of bacteria regulates various nutrients such as glucose non-specifically. However, modulation of certain nutrients such as triglyceride requires microbial interactions between two major endogenous bacteria, *Acetobacter* and *Lactobacillus* (Newell and Douglas, 2014; Sommer and Newell, 2019), highlighting the importance of microbial interactions. It is this simple model that makes comprehensive studies of the association of five major groups of endogenous gut bacteria with fly fitness traits experimentally possible. Emerging studies have revealed how the bacterial interactions support the key function often assigned to a major functional keystone species in terms of their impact on host physiology and pathology such as lifespan and reproduction of flies (**Figure 2**). The similar results and scenarios have been observed and reported in many other model systems (Chao et al., 2017), suggesting the simple fruit fly model can capture the key events from the complex interactions between host and the microbiome in highly diverse systems such as humans and rodent models. In addition, the recent finding of a symbiotic and persistent strain in the fly gut offers new insights into evolution and adaptation of host-microbe interactions. It is feasible to utilize flies as a model to farm microbes of interests to recapitulate human host-microbe interactions (Gould et al., 2018; Brinker et al., 2019). *In vitro* bacteria-bacteria interactions can be readily simulated in *Drosophila* by coinfection, which affords the identification of genetic determinants that are responsible for mutually beneficial or antagonizing interactions, and uncovers a metabolic basis for cross-feeding between bacterial metabolites and their impact on host fitness traits. Microbiota selectively acquired from the natural environment by *Drosophila* can modulate a wide variety of host cellular pathways and relevant physiology. Microbiota regulate flies’ hematopoietic pathway through a RUNX-like transcription factor, thereby affecting the fly development (Benoit et al., 2017). Recently an elegant study has illustrated the metabolic crosstalk between bacteria and fungi through ethanol catabolism and production of volatile compounds in flies. Metabolic cross feeding by co-culturing of *Saccharomyces* and *Acetobacter* triggers differential chemo-sensitive response and enhances egg-laying ability of flies via a conserved olfactory receptor, Or42b (Fischer et al., 2017), revealing a new link between polymicrobe-derived metabolites and host physiology. Olfaction is a crucial element through which flies detect exogenous cues and respond accordingly, which shapes host-microbe interactions including flies’ preference or avoidance for diverse microbes. It is worth noting that such preferences can vary depending on endogenous gut microbiome (Wong et al., 2017), which offers new avenues and opportunities to study dynamic microbiota, genes, and environmental interactions in a simple and tractable system. Another emerging area of studies in microbiome science and medicine is the investigation of brain-gut-microbiome axis in human health and disease (Dinan and Cryan, 2017; Schretter et al., 2018). It has been recognized that the gut has evolved to become a signal hub that integrates a wide range of inputs from different sources including complex microbial and microbe-host interactions. While there are some understanding of bidirectional communications between gut and brain, the underlying molecular mechanisms are poorly defined. The *Drosophila* is a simple and robust model system to investigate how microbial metabolites from microbial interactions contributes to brain-gut interactions and vice versa, which would offer unique new insights into microbiome-directed gut-brain axis and their contributions to our understanding of the role of microbiota in various neurological disorders such as anxiety, autism, depression or brain tumor. The *Drosophila* nervous system shares a high degree of similarity with its human counterpart, making it a suitable model for studying the molecular mechanisms underlying development, progression, and potential therapeutic targets of neurological disorders.

In addition, fruit flies can also serve as models for epithelial cancers, including colorectal cancers (CRC). Due to physiological and morphological similarities between fruit flies and mammals, the fly gut, particularly the midgut, is useful for studying the contributions of signaling pathways and metabolism in CRC (Jiang et al., 2022). Recurrent mutations in CRC have been modeled in flies, and multigenic fly models have been developed to unravel the complexity of CRC in terms of metabolism and drug response. These models have provided valuable insights into drug discovery and the functional exploration of human cancer genomes. Furthermore, personalized fly models enable high-throughput screening to develop targeted treatments for patients with refractory metastatic CRC. These studies have demonstrated the practicality of using flies in mechanistic analyses and drug discovery for CRC, highlighting the potential of *Drosophila* as a useful preclinical whole-animal model.

Given the key role of microbiota played in progression of CRC, notably *Fusobacterium nucleatum* (Bullman et al., 2017; Clay et al., 2022), it is desirable to employ the fruit flies model to systematically study interplay between microbiota and the development of CRC. This can serve as a feasible model to study the association of microbiota with other types of cancers including head & neck cancer.

The powerful genetic tools offered by the fly model also allows the study of how the residential gut microbiome evolved as a physical and immunological barrier that controls infection of other microbes such as enteric virus. Specific commensals are able to prime inflammatory responses that enables gut epithelial cells to restrict enteric viral infection of flies (Sansone et al., 2015). The fly model enables the rapid identification of various signals mediated by commensal and virus, which provides new perspectives into how dynamic interactions between microbiota and gut epithelial cells direct anti-infective immunity, an emerging exciting area of investigation.

Increasing studies using flies as a model have shown that commensal microbiome affects host aging and lifespan (Guo et al., 2014; Heintz and Mair, 2014; Lee et al., 2019). During the course of aging, the gut bacterial diversity and the overall abundance change substantially (Claesson et al., 2012; Nagpal et al., 2018; Aleman and Valenzano, 2019; Ragonnaud and Biragyn, 2021). Several studies have found these changes affect aging and lifespan in fruit flies (Guo et al., 2014; Clark et al., 2015; Lee et al., 2019). For instance, the shift in microbial composition in aging flies are associated with intestinal barrier failure and shortened lifespan (Clark et al., 2015). Alterations in the microbial composition precede and are linked to dysfunction of the gut epithelial barrier. The intestinal barrier failure in turn leads to a distinct shift in microbiota composition and causes systemic immune activation and the decline in organismal health and lifespan. These results are consistent with findings from an earlier study in which fly mutations that disrupt the septate junctions (SJs) of the gut epithelium induce higher immune activity and concurrently decrease lifespan (Bonnay et al., 2013). Feeding the mutant flies with antibiotics that ablate the commensal bacteria partially restore the lifespan of the mutant flies, showing the link between microbiota and fly lifespan. Additionally, oral infection with bacteria that are able to cross the intestinal barrier, including *Serratia marcescens DB11* and *Pseudomonas aeruginosa PA14*, significantly shorten lifespan of the SJs-disrupted flies, with an increased bacteria load in the hemolymph, indicating the importance of the gut epithelium in modulating microbial infection (Bonnay et al., 2013). Notably, the microbial composition changes are also observed in aged human gastrointestinal tract and in human inflammatory disorder patients (Claesson et al., 2011; Kim and Benayoun, 2020). This similarity further demonstrates *Drosophila* as one of the viable model organisms to investigate the interplay between microbial interactions, aging and lifespan.

## Limitations of the *Drosophila* model to study polymicrobial interactions

Despite the advantages of the use of the simple, inexpensive and high through-put fly model, it is crucial to recognize the existing limitations of the model. For instance, given the simple microbiota community harbored by flies, sometimes it is challenging to simulate complex microbe-microbe interactions that regulate human health and disease. Thus, careful experimental design is necessary to translate the findings from the *D. melanogaster* microbiota model (Douglas, 2018). It is well-known that flies lack a comprehensive adaptive immunity system mammals possess (Wong et al., 2016), thus the study of the microbiota and adaptive immunity link is not possible in the model, and some aspects of microbiota-influenced wound healing, tissue repairs and inflammation cannot be modeled. For instance, fibrosis and scarring cannot be readily recapitulated since specific types of cells or tissues responsible for the induction of fibrosis such as myofibroblasts and connective tissues don’t exist in flies. It is also critical to acknowledge the differences in gastrointestinal anatomy such as the absence of lamina propria from the *Drosophila* intestine, and overall physiology (Lemaitre and Miguel-Aliaga, 2013), which should help guide the design of appropriate studies to address relevant questions in the right context of host-microbe interactions. It is apparent that flies don’t have teeth, while we can mimic bacterial colonization and production of acids, two key events of cariogenesis responsible for the development of dental caries but there is no enamel surface that can be demineralized as evident in rodent animal models (Peng et al., 2016a). Thus, additional complementary models are needed to validate this particular feature in the pathogenesis. By the same token, it is necessary to confirm overall experimental findings from *Drosophila* using relevant rodent models of infection given the evolutionary divergence between flies and mammals.

## Conclusion and future directions

The *Drosophila* gut microbiome serves as an attractive model of low microbiome complexity. The question remains “Is it possible to establish relatively complex biofilm-like structure formed by polymicrobioal infections which frequently seen in humans and rodents given the simplicity of the endogenous microbial community in flies’ gut?” Another frequently asked question is whether some of the obligate anaerobes such as oral periodontal pathogens *P. gingivalis* and *T. denticola* could be incorporated as part of the polymicrobial community because the feeding model relies on the consumption of live bacteria by flies and anaerobes are very sensitive to oxygen killing. This may be circumvented by the use of alternative infection model, a pricking model, which bypasses the gut barriers, enabling inoculation of live bacteria and the study of other unique aspects of microbiome-host interactions (Igboin et al., 2011). In addition, by introducing the capillary feeding system (Murphy et al., 2017), one could constantly feed flies with freshly cultured bacteria and track the amounts of the bacteria the flies consume. It is well documented that dietary conditions, endogenous or invading microbial species, and innate host defense factors collectively determine successful rate of infection of flies by exogenous microbes, which provides the opportunities to establish polymicrobial colonization by taking all those factors into consideration. Such permutations of a wide range of environmental and dietary factors can be readily designed and implemented using the fly model while it would be too expensive to conduct in other systems. In this regard, taking advantage of the known polymicrobial synergy seen among different organisms should help facilitate the colonization of flies with a simple and functional polymicrobial community, in which anaerobes are supported by other microbes to become more resistant to aerobic conditions and then enhance the colonization of the community. This cost-effective, high throughput model makes it possible for the deconvolution of complex systems biology questions. As microbiome has emerged to be an important field in the study of human health and disease, validated fly models of human disease related to cardiovascular, immunological, and metabolic disorders could be readily explored to investigate the functional contributions of microbiota and host-microbe interactions to the development of these systemic conditions. The use of broad spectrum of antibiotics in childhood leads to dysbiosis that is linked to the development of chronic human disorders such as obesity, diabetes and other metabolic disorders, however, the underlying mechanisms are unknown. Epigenetic regulation triggered at the acquisition of dysbiotic microbiota during early life may prime the development of metabolic disorders (Thorburn et al., 2015; Gray et al., 2017), representing a potential microbial inheritable trait. The fly model is a robust system to assess bacterial inheritance, epigenetic regulation, and disease development in offer springs.

In summary, *Drosophila* is an excellent model for investigating mechanisms of microbe-microbe and host and microbe interactions. The simple microbial diversity makes it possible for developing advanced engineering tools, and techniques to facilitate the study of microbe-fly relationships using systems biology approaches, which should accelerate basic science discoveries and enable potential clinical translations.

## Author contributions

HW: conceptualization, data curation, formal analysis, funding acquisition, project administration, resources, software, supervision, validation, visualization, writing – original draft, writing – review & editing. XC: formal analysis, investigation, methodology, validation, writing – original draft, writing – review & editing. JS: conceptualization, formal analysis, writing – original draft, writing – review & editing. BX: investigation, methodology, writing – review & editing. DM: supervision, writing – review & editing.
